# Limb Salvage Versus Amputation: A Review of the Current Evidence

**DOI:** 10.7759/cureus.10092

**Published:** 2020-08-28

**Authors:** Mobeen K Qureshi, Ali Ghaffar, Sameem Tak, Ahmad Khaled

**Affiliations:** 1 Trauma and Orthopaedics, East Lancashire NHS Hospitals, Blackburn, GBR; 2 Trauma and Orthopaedics, Kettering General Hospital, Kettering, GBR

**Keywords:** limb salvage, amputation, trauma

## Abstract

In the trauma situation where the trauma team is faced with a severely injured limb, it requires judicious thinking and evaluating not only the injury in isolation but the patient as a whole when considering the management options. The aim must be to give the best quality of life and avoid repeated admissions to hospital for associated complications in the future.

The decision to amputate or salvage a limb should be based on numerous factors, such as the patient’s pre-injury status, injury factors (soft tissue injury, location, contamination and physiological status), patient’s wish and available resources.

The biggest challenge when faced with a complex limb injury is deciding what management route to take with a satisfactory outcome for the patient being the main goal.

Many studies have been undertaken looking at the outcome of successful limb salvage versus primary amputation. Studies such as the Lower Extremity Assessment Project (LEAP) study have concluded that there was no difference of outcome at the two-year stage between the two strategies.

## Introduction and background

In the trauma situation where the trauma team is faced with a severely injured limb, it requires judicious thinking and evaluating not only the injury in isolation but the patient as a whole when considering different management options. When considering whether to salvage a limb or to proceed with amputation, many variables must be taken into consideration; these include objective aspects, such as the injury and the comorbidities of the patient, as well the subjective aspects, such as the economic, social and psychological status. The aim must be to give the best quality of life and avoid repeated admissions to hospital for associated complications in the future. Currently, no consensus has been reached as how best to deal with these situations, as there have been few high-level studies.

Decision-making factors

The decision to amputate or salvage a limb should be based on numerous factors, such as the patient’s pre-injury status, injury factors such as soft tissue injury, location, contamination and physiological status, patient’s wishes and availability of resources [1].

The strategy of treatment options is based on the type of fracture and associated soft tissue damage, the duration of the warm ischaemic period and the overall condition of the patient [2]. In the case of polytrauma patients and in those who are in a critical condition physiologically, limb salvage may be contraindicated, as the priority is to preserve life [3]. In patients who are stable but have a severely injured limb, the decision for limb salvage or amputation primarily depends on the level of soft tissue injury sustained, as well as neurovascular damage impacting the long-term outcomes. When considering the vascular status, it has been shown that a warm ischaemia time of greater than six hours greatly reduces the success rate of a salvage procedure [4]. However, this can be improved if the limb is in ice-cold water for four to eight hours, giving a window of up to eight hours to perform a salvage procedure [5]. In those with a nerve injury, some studies have favoured amputation such as for a Gustilo type IIIC fracture with associated nerve injury [6]. Another study recommended amputation for patients with posterior tibial nerve impairment [4]. For injuries with soft tissue involvement, the key factor that will determine a good outcome is the coverage with an adequate blood supply, which will prevent the risk of infections and non-union. Another determinant is adequate debridement of necrotic tissue.

Another consideration is the level of amputation. The goal is to preserve as much of the limb as possible. Using the lower limb as an example, a long limb uses less energy when walking; therefore, a below-knee amputation is preferable to through-the-knee and above-knee amputations [7].

The wish of the patient should also be considered when making a decision. Some patients may not be able to afford a protracted absence from employment and therefore may decide to have an amputation. Other personal factors include social support, availability of rehabilitation services and the willingness to engage with such services.

Depending on the medical system within which a patient is treated, the financial cost may dictate the decision-making process. The decision to salvage a limb that eventually leads to amputation will be a costlier decision. Goldberg et al. calculated the cost of hospitalization per patient to be $18,698, of which patients paid $2,261 on average in the United States of America [8]. Clearly a burden on low income individuals and their family. However, another study has concluded that amputation is costlier than limb salvage. Chung et al. stated that an amputation for a 40-year-old patient will cost at least $93,606 and up to $154,636 more than limb salvage (independent of ongoing prosthesis needs) [9]. In a state-funded healthcare system, the financial aspect would not have impact on the patient directly.

It has also been shown that delayed amputation carries an increased risk of sepsis and infective complication as compared to primary amputation [10].

A multitude of scoring systems have been developed to aid the surgeon when evaluating the severity of an injured limb. These include the Mangled Extremity Severity Index (MESI) devised by Gregory et al., which is further developed by McNamara et al. into the Nerve injury, Ischemia, Soft-tissue contamination, Skeletal injury, Shock and Age (NISSA) score, Hanover Fracture Scale (HFS) and the Limb Salvage Index (LSI) [6,11-13]. However, these scoring systems have been shown to be imprecise when prospectively making a clinical decision whether to amputate or salvage a limb [14-16]. This is mainly thought to be due to the scores being devised from retrospective studies, as well as other factors such as differences in an interobserver grouping of the severely injured limb.

A recent retrospective study carried out looked at 1,354 patients with mangled lower extremities who were treated at 222 level I and II trauma centres. Only 21% of patients underwent amputation, with half of these amputations occurring within 24 hours of the injury. Amputation was most likely in those with certain types of injury, high energy impact, associated injures such as severe head injury and hypotension [17]. This study suggests that the majority of injured limbs are being salvaged and that amputation only occurs in the immediate period, most likely due to the inevitability that an amputation will occur at some stage.

## Review

Outcomes

The biggest challenge when faced with a complex limb injury is deciding what management route to take with a satisfactory outcome for the patient being the main goal. As a consequence, the medical team has to consider different factors, such as the long-term psychological impact, functional and quality of life (QoL) outcomes. There has been much debate on how to define and measure QoL [18]. However, a consensus points to a range of domains, such as physical, psychological and social.

Patient factors also dictate the outcome and therefore together with physical, social and psychological domains, economic status must be considered. Francel reported that patient factors rather than the severity of the injury correlated with a successful return to work. Patients who were younger than 40 years, more educated and in white-collar employment tended to fare better [19].

In 2006, Barros et al. published “The Belfast Approach,” which advocates the early placement of shunts in both artery and vein, with the aim of reconstruction of all injured anatomical structures on the first encounter. They state that the shunt ultimately buy time for meticulous care, significantly reducing fasciotomies and resulting in a significant reduction in the incidence of contracture and amputation. They also championed the case for optimal vein reconstruction. This strategy was shown to reduce the incidence of re-exploration and secondary repair [20]. 

Hoogendoorn and van der Werken looked at grade III open tibial fractures and the functional outcome and QoL in amputees versus patients with successful reconstruction. They concluded that patients with successful reconstruction experience significantly more complications, requiring further surgical interventions, as compared to those who underwent primary amputation. The complications related to the former group were due to problems in bone healing [21].

This is countered by Dagum et al. who have reported that patients who underwent amputation had lower physical functional outcome scores compared to those who had successful limb salvage procedures [22]. In another study, Puno et al. reported no difference in function and pain between primary amputation and limb salvage [23].

According to MacKenzie et al., predictors of poor outcomes include rehospitalizations for a major complication, non-white race, lower socioeconomic status, low self-efficacy, smoking and involvement in disability compensation litigation [24]. These findings are difficult to implement and consider when making a clinical decision.

The highest level of evidence available was conducted by Bosse et al. and was named the Learning Early About Peanut Allergy (LEAP) study. It was a multicentre prospective cohort study including 601 patients enrolled from eight level I trauma centres in the United States of America who had sustained high-energy lower limb injuries. The primary outcome of the study was derived using a self-reported measure of health status that was called the Sickness Impact Profile (SIP), which was measured for several months after the injury. Scored ranged from 0 to 100 with a score >10 considered to represent severe disability [25]. The conclusion reached by the study was that at the two-year follow-up point there was no difference in the health status between those who had undergone amputation compared to those who had had their limb salvaged. It was noted however that those who underwent amputation were less likely to require rehospitalizations.

A meta-analysis was conducted by the Evidence-Based Orthopaedic Trauma Working Group in 2007 that also supported the LEAP study by concluding that there was no significant difference of functional outcome in those treated with either an amputation or successful limb salvage, at least up to seven years post-injury [26].

Another meta-analysis published in 2011 by Akula et al. concluded that lower limb reconstruction is more acceptable psychologically to patients with severe lower limb trauma compared to amputation; however, physical outcomes for both management strategies were similar [27].

In military studies, from the experience in Afghanistan and Iraq, data have been utilized from the Expeditionary Medical Encounter Database. In a retrospective study from 2001 to 2008, there were a total of 587 early amputees and 117 limb salvage patients. It was shown that early amputees had similar physical outcomes but were noted to have a lower rate of psychological disorders [28].

## Conclusions

The decision to either amputate or salvage a limb is a complex one. There is a multitude of factors that dictate not only the decision but also the outcome. When looking at the decision-making progress, different aspects such as environmental factors will influence which route to take. There will inevitably be a difference depending on the location of the patient. If they live in an area that has no established trauma network or in a rural area that does, then the availability of specialists will dictate the decision. The status of a patient is also crucial, as in the case of a polytrauma patient who is critical, the main goal would be to preserve life and not necessarily save the limb. In patients who are fortunate to be bought to a trauma centre with all the available specialists, then a multidisciplinary approach must be taken. When making a decision, if possible the patient must be involved in the process. In private healthcare systems, the financial burden for the patient and immediate family may influence what can and cannot be done.

As has been shown many studies have been undertaken looking at the outcome of successful limb salvage versus primary amputation. A consensus has not been reached and many studies argue one strategy over the other; however, the higher level of evidence has become available over the last decade including the LEAP study, which concluded that there was no difference at the two-year stage between the two strategies. Two meta-analyses have given similar conclusions, with reconstructive surgery being suggested as the better strategy based only on better psychological outcomes. This does come at a risk of greater rehospitalizations. This all leads to the crucial step of involving the patient in the decision process. This in itself can be challenging as in the critical period post trauma, a patient may not be able to communicate or be able to give an informed decision.
