# Correction: Limb Salvage Versus Amputation: A Review of the Current Evidence

**DOI:** 10.7759/cureus.c38

**Published:** 2020-10-07

**Authors:** Mobeen K Qureshi, Ali Ghaffar, Sameem Tak, Ahmad Khaled

**Affiliations:** 1 Trauma and Orthopaedics, East Lancashire NHS Hospitals, Blackburn, GBR; 2 Orthopaedics and Trauma, East Lancashire NHS Hospitals, Blackburn, GBR; 3 Trauma and Orthopaedics, Kettering General Hospital, Kettering, GBR

During final copy editing of this article, the acronym LEAP was incorrectly expanded to "Learning Early About Peanut Allergy" instead of the correct "Lower Extremity Assessment Project" in the article abstract. Cureus sincerely regrets this error and wishes to make clear that the authors were in no way responsible for this error. The text has been corrected in the article.

